# Supplementary material for: Exploring the awareness, attitudes, and actions (AAA) of UK adults at high risk of severe illness from COVID-19
Source: PLoS One. 2021 Nov 11;16(11):e0259376. doi: 10.1371/journal.pone.0259376 (PMC8584659; doi:10.1371/journal.pone.0259376)
Supplement: S1 File — (DOCX) [file pone.0259376.s001.docx]

**S1 Supplementary materials: Awareness, Attitudes and Actions (AAA) survey**

| **Survey questions** | **Response categories/instruction** |
| --- | --- |
| ***Section A: demographics*** | |
| Does any of the following apply to you? (select all that apply) | Diabetes (Type 1 or 2)  A body mass index (BMI) of  40 or above  Chronic (long-term) respiratory diseases, such as asthma, chronic obstructive pulmonary disease (COPD), emphysema or bronchitis  Chronic heart disease, such as heart failure  Chronic kidney disease  Chronic liver disease, such as hepatitis  Chronic neurological conditions, such as Parkinson’s disease, motor neurone disease, multiple sclerosis (MS), a learning disability or cerebral palsy  Problems with your spleen – for example, sickle cell disease or if you have had your spleen removed  A weakened immune system as the result of conditions such as HIV and AIDS, or medicines such as steroid tablets or chemotherapy  None of these apply to me  I have a different long term health condition not listed above (please specify in the text box provided) |
| Please state your age | Textbox |
| Gender | Male  Female  Other (textbox)  Prefer not to say |
| What is your ethnicity? | White – British, Irish, other  Asian/Asian British – Indian, Pakistani, Bangladeshi, other  Chinese/Chinese British  Black/Black British – Caribbean, African, other  Middle Eastern/Middle Eastern British – Arab, Turkish, other  Mixed race – White and Black/Black British  Mixed race – other  Other ethnic groups (please specify in the text box provided)  Prefer not to say |
| What is your height in feet and inches, or centimetres? | Text box provided for each |
| What is your weight in pounds or kilograms? | Text box provided for each |
| Do you work in health or social care? | Yes (please provide your job title in the text box)  No |
| Does your job require you to be in direct contact with coronavirus (COVID-19) patients? | Yes  No |
| Please provide the first half of your postcode (e.g. NG1) | Textbox provided |
| Please provide your email address | Textbox provided |
| ***Section B: awareness, attitudes and actions relating to COVID-19*** | |
| Have you had coronavirus? | Yes – I have been diagnosed and am still ill  Yes – I have and I have recovered  Yes - I have been diagnosed, but had no symptoms  No |
| Have you experienced coronavirus symptoms? | Yes - and I was diagnosed  Yes – but I have not been diagnosed  No  I don’t know what the symptoms |
| Which of the below are symptoms of coronavirus? (Select all that is relevant) | Persistent cough  Feeling confused  Loss of appetite  Loss of smell  Loss of taste  Tightness in chest  Diarrhoea  Fatigue  Shortness of breath  Fever  Sore throat  None of the above |
| Have you taken any of the actions below in response to the coronavirus (COVID-19) outbreak? (select all that apply) | Social distancing  Self-isolation  Worn protective apparel (e.g. gloves, mask etc.)  Used online shopping or food delivery service  Shielding due as my health status means I am defined as 'extremely vulnerable'  All of the above  Other (Textbox) |
| Do you believe you are at higher risk of severe illness from coronavirus (COVID-19)? | Yes  No |
| Why do you believe you are at a higher risk of severe illness from coronavirus (COVID-19)? (only for those who answered yes) | Textbox |
| Why do you believe you are not at a higher risk of severe illness from coronavirus (COVID-19)? (only for those who answered no) | Textbox |
| Describe how being identified as being at a higher risk of severe illness from coronavirus (COVID-19) by the UK Government, has made you feel? | Textbox |
| What sources have informed you that you are at a higher risk from coronavirus (COVID-19)? (select all that apply) | Traditional media (TV, Newspapers, Radio)  Social media (Twitter, Facebook, Instagram, Snapchat)  National or Local Government  Employer  Healthcare organisations  Community groups  Charity  Friends and Family  Schools and education centres  Other (please specify in the text box provided) |
| Do you feel like you have enough information specific to your higher risk of severe illness from coronavirus (COVID-19)? | Yes  No |
| Why do you believe you have received enough information specific to your higher risk of severe illness from coronavirus (COVID-19), and what more do you want to know? (only for those who answered yes) | Textbox |
| Why do you believe you have not received enough information specific to your higher risk of severe illness from coronavirus (COVID-19), and what else do you want to know? (only for those who answered no) | Textbox |
| Have you used other forms of information (i.e. nonprofessional/social media “experts”/other people/patients) since the COVID-19 outbreak? | Yes  No |
| Please specify what information you have used relating to your higher risk status since the coronavirus (COVID19) outbreak | Textbox |
| How concerned are you about each of the statements below   - Becoming infected with coronavirus (COVID-19) - Severe illness and possibly death from coronavirus (COVID-19) - Spreading coronavirus (COVID-19) to others including family and friends - Access to healthcare support (e.g. advice, medication) - If you become infected, that you would receive appropriate care/support - That your higher risk of severe illness from coronavirus (COVID-19) means you may not receive healthcare support compared with people who do not have a higher risk status | Likert scale from 0 (Not concerned at all) to 10 (Very concerned) |
| ***Section C: impact of COVID-19 on management of health conditions and use of technology*** | |
| Has your management of your health condition changed compared to before the coronavirus (COVID-19) outbreak? | Yes  No  Not applicable (70 years or over or pregnant without a health condition) |
| How and why has it changed? | Textbox |
| How do you feel about changing your management of your health condition due to the coronavirus (COVID-19) outbreak? | Textbox |
| Has COVID-19 changed your regular healthcare support? (this could type or frequency of support e.g. appointments, service, medications, communication consultant) | Appointments (please specify in the text box)  Medication (please specify in the text box)  Elective surgery (please specify in the text box)  Communication platform (please specify in the text box)  Clinician caring for me (please specify in the text box)  Other (please specify in the text box)  There has been no change |
| Have you received care through any of the following platforms? | Social media (please specify in the text box)  Mobile phone app (please specify in the text box)  Email  Telephone Virtual consultation e.g. Zoom, Microsoft Teams (please specify in the text box)  Other (please specify in the text box)  No platforms have been used  I am still receiving face to face care |
| How satisfied are you with using the platforms that you are receiving care through? | Extremely dissatisfied  Somewhat dissatisfied  Neither satisfied nor dissatisfied  Somewhat satisfied  Extremely satisfied |
| How satisfied are you with using the information/resources provided through the platforms that you are receiving care through? | Extremely dissatisfied  Somewhat dissatisfied  Neither satisfied nor dissatisfied  Somewhat satisfied  Extremely satisfied |
| Would you welcome the continued use of these platforms in the future, after the coronavirus (COVID-19) outbreak? | Yes  No, but would welcome other platforms (please specify in the text box)  No  Not sure, I need more time to use them |
| You indicated that you have more than one of the high risk indicator for severe illness from coronavirus (COVID19). Please describe how this makes you feel, and why? | Textbox |
| ***Section D: Mental Health and Wellbeing*** | |
| Since the coronavirus (COVID-19) outbreak, my mental health is | Yes  No |
| Warwick-Edinburgh Mental Well-being Scale (WEMWBS)  During the past two weeks...   - I’ve been feeling optimistic about the future - I’ve been feeling useful - I’ve been feeling relaxed - I’ve been feeling interested in other people - I’ve had energy to spare - I’ve been dealing with problems well - I’ve been thinking clearly - I’ve been feeling good about myself - I’ve been feeling close to other people - I’ve been feeling confident - I’ve been able to make up my own mind about things - I’ve been feeling loved - I’ve been interested in new things - I’ve been feeling cheerful | Not at all  Rarely  Some of the time  Often  All of the time |
| Patient Health Questionnaire (PHQ-9)  Over the last two weeks, how often have you been bothered by any of the following problems   - Little interest or pleasure in doing things? - Feeling down, depressed, or hopeless? - Trouble falling or staying asleep, or sleeping too much? - Feeling tired or having little energy? - Poor appetite or overeating? - Feeling bad about yourself - or that you are a failure or have let yourself or your family down? - Trouble concentrating on things, such as reading the newspaper or watching television? - Moving or speaking so slowly that other people could have noticed? Or the opposite - being so fidgety or restless that you have been moving around a lot more than usual? - Thoughts that you would be better off dead, or of hurting yourself in some way? | Not at all  Several days  More than half the days  Nearly every day |
|  |  |
| ***Section D: lifestyle related behaviours*** |  |
| Has your shopping changed since the coronavirus (COVID-19) outbreak? | A great deal  A lot  A moderate amount  A little  Not at all |
| Describe how your shopping has changed since the coronavirus (COVID-19) outbreak | Textbox |
| Has your diet changed since the coronavirus (COVID19) outbreak? | A great deal  A lot  A moderate amount  A little  Not at all |
| Describe how your diet has changed since the coronavirus (COVID-19) outbreak | Textbox |
| Has your alcohol consumption changed since the coronavirus outbreak? | I have consumed much less alcohol than usual  I have consumed less alcohol than usual  It hasn't changed  I have consumed more alcohol than usual  I have consumed much more alcohol than usual |
| Why has your alcohol consumption changed since the coronavirus (COVID-19) outbreak? | Textbox |
| Has the amount of physical activity you usually engage in changed since the coronavirus outbreak? | I am much less active  I am less active  It hasn't changed  I am more active  I am much more active |
| Has the type of physical activity you usually engage in changed since the coronavirus outbreak? | Yes  No |
| Describe how and why your physical activity has changed since the coronavirus outbreak | Textbox |
| Has the amount or quality of your sleep changed since the coronavirus outbreak? | A great deal  A lot  A moderate amount  A little  Not at all |
| Describe how and why the amount or quality of your sleep has changed since the coronavirus outbreak | Textbox |
| Do you smoke tobacco? | Yes  No |
| Has the amount of tobacco you smoke changed compared to before the coronavirus (COVID-19) outbreak? | Much more  Somewhat more  About the same  Somewhat less  Much less |
| Do you use e-cigarettes? | Yes  No |
| Has the amount of e-cigarettes you use changed compared to before the coronavirus (COVID19) outbreak? | Much more  Somewhat more  About the same  Somewhat less  Much less |
| Other than alcohol or tobacco, do you use any recreational drugs? | Yes  No |
| Has the amount of recreational drugs you use changed compared to before the coronavirus (COVID19) outbreak? | Much more  Somewhat more  About the same  Somewhat less  Much less |
| ***Section E: Interaction with others*** |  |
| For the following questions, please respond with your health condition or higher risk status (70 years old or over or pregnant regardless of medical conditions) in mind. Since the coronavirus (COVID-19) outbreak... | |
| Other people have behaved differently towards you? | Yes  No |
| Describe how and why people have behaved differently towards you since the COVID-19 outbreak? | Textbox |
| You felt stigmatised or discriminated against? | Yes  No |
| Describe the stigmatising and/or discriminatory experience(s) you have had since the COVID-19 outbreak, and how this has made you feel? | Textbox |
| ***Final section*** | |
| Is there anything that you haven't had chance to say about the coronavirus outbreak that you would like to share? | Textbox |

**Data from the open-ended questions highlighted in red font were analysed in this study*
