# Supplementary material for: Exploring the awareness, attitudes, and actions (AAA) of UK adults at high risk of severe illness from COVID-19
Source: PLoS One. 2021 Nov 11;16(11):e0259376. doi: 10.1371/journal.pone.0259376 (PMC8584659; doi:10.1371/journal.pone.0259376)
Supplement: Attachment — Submitted filename: plos one rev UK AAA covid 19 2021.docx [file pone.0259376.s003.docx]

Dear Authors,

Thanks for submitting your article to PlosOne. Thank you for giving me the opportunity to read your very interesting work.

The subject of this article is impact of COVID-19 lockdown in a high risk of severe illness UK patients. It is a relevant subject. Authors used a specific questionnaire described in a previous article (BMJ Open 2020). This is a qualitative study on answers from this survey. Methods are relevant. Few data on this field have been published. Tables and results are well described. The article is well structured.

Aims of the study are clearly written in introduction and answers well described in discussion and conclusion

English language is good.

Minor revisions:

In Introduction p 4 l76-78: introduction or methods. I think it is relevant to have a specific sentence on your definition of the term impact in methods.

Table 1: in the part attitudes and concerns and also self-isolation be careful on tabulation

Defined +ve and – ve in legend

P 13 l 261 put a ref (maybe not scientific) on Dominic Cumming’s incident or explain it in some words for non UK readers…

Discussion:

Shortened the discussion

Some ref could be added as:

[Psychological distress during the COVID-19 pandemic in France: a national assessment of at-risk populations.](https://pubmed.ncbi.nlm.nih.gov/34192239/)

Chaix B, Delamon G, Guillemassé A, Brouard B, Bibault JE. Gen Psychiatr. 2020 Nov 26;33(6):e100349. doi: 10.1136/gpsych-2020-100349. eCollection 2020.

PMID: 34192239 Free PMC article.

[The limitations of polling data in understanding public support for COVID-19 lockdown policies.](https://pubmed.ncbi.nlm.nih.gov/34258021/)

Foad CMG, Whitmarsh L, Hanel PHP, Haddock G. R Soc Open Sci. 2021 Jul 7;8(7):210678. doi: 10.1098/rsos.210678. eCollection 2021 Jul.

PMID: 34258021 Free PMC article.

| 1. | [Understanding the psychological impact of the COVID-19 pandemic and containment measures: An empirical model of stress.](https://pubmed.ncbi.nlm.nih.gov/34324498/)  Wissmath B, Mast FW, Kraus F, Weibel D.  PLoS One. 2021 Jul 29;16(7):e0254883. doi: 10.1371/journal.pone.0254883. eCollection 2021.  PMID: 34324498 Free PMC article. |
| --- | --- |
| 2. | [Impact of COVID-19-like symptoms on occurrence of anxiety/depression during lockdown among the French general population.](https://pubmed.ncbi.nlm.nih.gov/34310661/)  Mary-Krause M, Herranz Bustamante JJ, Héron M, Andersen AJ, El Aarbaoui T, Melchior M.  PLoS One. 2021 Jul 26;16(7):e0255158. doi: 10.1371/journal.pone.0255158. eCollection 2021.  PMID: 34310661 Free PMC article. |
