# Supplementary material for: Exploring the awareness, attitudes, and actions (AAA) of UK adults at high risk of severe illness from COVID-19
Source: PLoS One. 2021 Nov 11;16(11):e0259376. doi: 10.1371/journal.pone.0259376 (PMC8584659; doi:10.1371/journal.pone.0259376)
Supplement: Attachment — Submitted filename: Response to reviewers_comments_PLOS ONE.docx [file pone.0259376.s004.docx]

**Journal and Reviewers comments**

**Thank you very much for the editors and reviewers useful comments which we have addressed them below. Please find our responses in bold.**

1. Please ensure that your manuscript meets PLOS ONE's style requirements, including those for file naming. The PLOS ONE style templates can be found at <https://journals.plos.org/plosone/s/file?id=wjVg/PLOSOne_formatting_sample_main_body.pdf> and

[https://journals.plos.org/plosone/s/file?id=ba62/PLOSOne_formatting_sample_title_authors_affiliations.pdf](https://eur01.safelinks.protection.outlook.com/?url=https%3A%2F%2Fjournals.plos.org%2Fplosone%2Fs%2Ffile%3Fid%3Dba62%2FPLOSOne_formatting_sample_title_authors_affiliations.pdf&data=04%7C01%7Ca.c.brown%40ucl.ac.uk%7Cde1f9a2363c04396a79f08d95bfce602%7C1faf88fea9984c5b93c9210a11d9a5c2%7C0%7C0%7C637641965403193354%7CUnknown%7CTWFpbGZsb3d8eyJWIjoiMC4wLjAwMDAiLCJQIjoiV2luMzIiLCJBTiI6Ik1haWwiLCJXVCI6Mn0%3D%7C1000&sdata=ImKz5N4v5HwTLAL1SCyfYtM%2FcH5KtfE9pvwLJrrCARI%3D&reserved=0)

**Thank you for your comment we have reviewed the PLOS ONE style requirements and amended accordingly.**

2. We note that the grant information you provided in the ‘Funding Information’ and ‘Financial Disclosure’ sections do not match.

When you resubmit, please ensure that you provide the correct grant numbers for the awards you received for your study in the ‘Funding Information’ section.

**Thank you for the comment this has been added to the Funding information section as requested as follows:**

***“This study was partly supported by an investigator-initiated grant from Ethicon, Johnson & Johnson to SWF; the funder had no role in study design, data analysis, data interpretation, or writing of the report (grant number: 202004001)”***

3. Thank you for stating the following in the Acknowledgments Section of your manuscript:
“This study was partly supported by an investigator-initiated grant from Ethicon, J&J; the funder had no role in study design, data analysis, data interpretation, or writing of the report.”

We note that you have provided funding information that is not currently declared in your Funding Statement. However, funding information should not appear in the Acknowledgments section or other areas of your manuscript. We will only publish funding information present in the Funding Statement section of the online submission form.
Please remove any funding-related text from the manuscript and let us know how you would like to update your Funding Statement. Currently, your Funding Statement reads as follows:
 “This study was partly supported by an investigator-initiated grant from Ethicon, J&J to SWF; the funder had no role in study design, data analysis, data interpretation, or writing of the report.”

Please include your amended statements within your cover letter; we will change the online submission form on your behalf.

**This has been removed from the main text and has been added to the reply letter as follows:**

***“This study was partly supported by an investigator-initiated grant from Ethicon, Johnson & Johnson to SWF; the funder had no role in study design, data analysis, data interpretation, or writing of the report (grant number: 202004001)”***

4. In your Data Availability statement, you have not specified where the minimal data set underlying the results described in your manuscript can be found. PLOS defines a study's minimal data set as the underlying data used to reach the conclusions drawn in the manuscript and any additional data required to replicate the reported study findings in their entirety. All PLOS journals require that the minimal data set be made fully available. For more information about our data policy, please see <http://journals.plos.org/plosone/s/data-availability>.

"Upon re-submitting your revised manuscript, please upload your study’s minimal underlying data set as either Supporting Information files or to a stable, public repository and include the relevant URLs, DOIs, or accession numbers within your revised cover letter. For a list of acceptable repositories, please see <http://journals.plos.org/plosone/s/data-availability#loc-recommended-repositories>. Any potentially identifying patient information must be fully anonymized.

Important: If there are ethical or legal restrictions to sharing your data publicly, please explain these restrictions in detail. Please see our guidelines for more information on what we consider unacceptable restrictions to publicly sharing data: <http://journals.plos.org/plosone/s/data-availability#loc-unacceptable-data-access-restrictions>. Note that it is not acceptable for the authors to be the sole named individuals responsible for ensuring data access.

We will update your Data Availability statement to reflect the information you provide in your cover letter.

**Thank you for your comment. We have added the study minimal data set to the resubmission find attached.** **The qualitative data within the study offers in some instances personal and sensitive experiences during COVID-19 e.g., about personal clinical experiences with clinician and about medical conditions, which could result in people being identified. But can be made available for researchers who meet the criteria for access to confidential data on request to the School of Psychology Research ethic committee University of Leeds**

**We have changed the data sharing statement as recommended to the following:**

**"The datasets used in the current study cannot be shared publicly because they contain potentially sensitive and identifiable patient information. Data are available on request from the University of School of Psychology Research ethic committee University of Leeds for researchers who meet the criteria for access to confidential data (psyc-ethicssubmissions@leeds.ac.uk)"**

5. We note that you have stated that you will provide repository information for your data at acceptance. Should your manuscript be accepted for publication, we will hold it until you provide the relevant accession numbers or DOIs necessary to access your data. If you wish to make changes to your Data Availability statement, please describe these changes in your cover letter and we will update your Data Availability statement to reflect the information you provide.

**As above, we have added the study minimal data set to the resubmission find attached.**

6. Please note that in order to use the direct billing option the corresponding author must be affiliated with the chosen institute. Please either amend your manuscript to change the affiliation or corresponding author, or email us at [plosone@plos.org](mailto:plosone@plos.org) with a request to remove this option.

**Dr Adrian Brown is affiliated with the University College London and should be the corresponding author. There was no place within the revision section of journal platform to confirm this. But have indicated on the manuscript that Dr Brown is the corresponding author and submitted the manuscript.**

Please review your reference list to ensure that it is complete and correct. If you have cited papers that have been retracted, please include the rationale for doing so in the manuscript text, or remove these references and replace them with relevant current references. Any changes to the reference list should be mentioned in the rebuttal letter that accompanies your revised manuscript. If you need to cite a retracted article, indicate the article’s retracted status in the References list and also include a citation and full reference for the retraction notice.

**Thank you for your comment. We have checked the referencing and added further references in response to changes to the reviewers comments.**

7. Thank you for submitting the above manuscript to PLOS ONE. During our internal evaluation of the manuscript, we found significant text overlap between your submission and the following previously published works, some of which you are an author.

<https://bmjopen.bmj.com/content/10/12/e045309.info>

We would like to make you aware that copying extracts from previous publications, especially outside the methods section, word-for-word is unacceptable. In addition, the reproduction of text from published reports has implications for the copyright that may apply to the publications.

Please revise the manuscript to rephrase the duplicated text, cite your sources, and provide details as to how the current manuscript advances on previous work. Please note that further consideration is dependent on the submission of a manuscript that addresses these concerns about the overlap in text with published work.

We will carefully review your manuscript upon resubmission, so please ensure that your revision is thorough.

**Thank you for your feedback and for noting that these sections needed to be addressed in particular in the introduction and methods. We have significantly revised the text to avoid any overlap with the previous publication, please see update manuscript.**

1. Please include captions for your Supporting Information files at the end of your manuscript, and update any in-text citations to match accordingly. Please see our Supporting Information guidelines for more information: <https://eur01.safelinks.protection.outlook.com/?url=http%3A%2F%2Fjournals.plos.org%2Fplosone%2Fs%2Fsupporting-information&amp;data=04%7C01%7Ca.c.brown%40ucl.ac.uk%7C35cc163fee454a82452408d981f1e66a%7C1faf88fea9984c5b93c9210a11d9a5c2%7C0%7C0%7C637683698214095605%7CUnknown%7CTWFpbGZsb3d8eyJWIjoiMC4wLjAwMDAiLCJQIjoiV2luMzIiLCJBTiI6Ik1haWwiLCJXVCI6Mn0%3D%7C1000&amp;sdata=%2Bo0eMgfOiavaUpH47CermovLzFfv5Y5m5VUoayEKL3I%3D&amp;reserved=0>.

**Thank you for the comment. We have now included a caption at the end of the manuscript for the supplementary materials called: S1 Supplementary Information: Awareness, Attitudes and Actions (AAA) survey. This is identified within the text where needed.**

**Reviewer 1:**

**We thank this Reviewer for their helpful comments regarding our manuscript and we have addressed each point below.**

My answer is generally very favorable, but one point needs clarification. The author repeatedly associates "mental health" and "well-being". So there seems to be a closeness between the two terms as well as a difference. Could he be more specific?

**Thank you for your useful and insightful comment. Mental health and wellbeing are interrelated terms. Mental health is defined as “a positive concept related to the social and emotional wellbeing of individuals and communities”. Wellbeing can be wider to include physical, social as well as psychological wellbeing. Therefore we have used the term “mental health and wellbeing” to be inclusive of both concepts.**

**Reviewer 2:**

**We thank this Reviewer for their helpful comments regarding our manuscript and we have addressed each point below.**

Dear Authors,

Thanks for submitting your article to PlosOne. Thank you for giving me the opportunity to read your very interesting work.

The subject of this article is impact of COVID-19 lockdown in a high risk of severe illness UK patients. It is a relevant subject. Authors used a specific questionnaire described in a previous article (BMJ Open 2020). This is a qualitative study on answers from this survey. Methods are relevant. Few data on this field have been published. Tables and results are well described. The article is well structured.

Aims of the study are clearly written in introduction and answers well described in discussion and conclusion

English language is good.

Minor revisions:

In Introduction p 4 l76-78: introduction or methods. I think it is relevant to have a specific sentence on your definition of the term impact in methods.

**Thank you for your comment we have moved the sentence within the introduction to the methods and clarified how we are defining impact.**

Table 1: in the part attitudes and concerns and also self-isolation be careful on tabulation

**Thank you for the comment we have reviewed the table and the column and rows appear to be appropriately formatted. But we have add addition lines to help**

Defined +ve and – ve in legend

**Thank you we have added in this to the note page line**

P 13 l 261 put a ref (maybe not scientific) on Dominic Cumming’s incident or explain it in some words for non UK readers…

***Thank you for your comment we have clarified who Dominic Cumming was and also referenced a correspondence published in the Lancet on the incident.***

Discussion:

**Shortened the discussion**

**Thank you for your comment. We have reviewed the discussion and shortened the discussion. Please see revised manuscript.**

Some ref could be added as:

**Thank you for your recommendation for adding references we have reviewed them and added two of the suggested recommendations that were appropriate.**

[Psychological distress during the COVID-19 pandemic in France: a national assessment of at-risk populations.](https://pubmed.ncbi.nlm.nih.gov/34192239/)

Chaix B, Delamon G, Guillemassé A, Brouard B, Bibault JE. Gen Psychiatr. 2020 Nov 26;33(6):e100349. doi: 10.1136/gpsych-2020-100349. eCollection 2020.

[The limitations of polling data in understanding public support for COVID-19 lockdown policies.](https://pubmed.ncbi.nlm.nih.gov/34258021/)

Foad CMG, Whitmarsh L, Hanel PHP, Haddock G. R Soc Open Sci. 2021 Jul 7;8(7):210678. doi: 10.1098/rsos.210678. eCollection 2021 Jul.
